# Association of Noise Exposure, Genetic Susceptibility, and Lifestyle With Type 2 Diabetes: A Prospective Cohort Study

**DOI:** 10.1155/jdr/8974749

**Published:** 2025-11-18

**Authors:** Dongming Wang, Luowen Zhou, Xingjie Hao, Zhaomin Chen, Bing Wang, Wenzhen Li

**Affiliations:** ^1^Department of Occupational & Environmental Health, School of Public Health, Tongji Medical College, Huazhong University of Science and Technology, Wuhan, Hubei, China; ^2^Key Laboratory of Environment and Health, Ministry of Education & Ministry of Environmental Protection, and State Key Laboratory of Environmental Health (Incubating), School of Public Health, Tongji Medical College, Huazhong University of Science and Technology, Wuhan, Hubei, China; ^3^Key Laboratory of Radiation Environment & Health of the Ministry of Ecology and Environment, China Institute for Radiation Protection, Taiyuan, China; ^4^Department of Neurosurgery, Tongji Hospital, Tongji Medical College, Huazhong University of Science and Technology, Wuhan, Hubei, China; ^5^Department of Epidemiology and Biostatistics, School of Public Health, Tongji Medical College, Huazhong University of Science and Technology, Wuhan, Hubei, China; ^6^Department of Reproductive Medicine, The Second People's Hospital of Yibin, Yibin, Sichuan, China; ^7^Jockey Club School of Public Health and Primary Care, The Chinese University of Hong Kong, Hong Kong, China; ^8^Shenzhen Research Institute of the Chinese University of Hong Kong, Shenzhen, China

**Keywords:** genetic susceptibility, lifestyle, noise exposure, T2D, workplace

## Abstract

**Aims:**

We aimed to explore the relationship of noise exposure in the workplace, genetic risk, and lifestyle with Type 2 diabetes (T2D).

**Methods:**

A total of 154,708 participants without T2D in UK Biobank were included. A lifestyle score was determined using smoking, alcohol intake, physical activity, television viewing time, sleep duration, and diet.

**Results:**

During a median follow-up of 11.83 years (1,776,919.62 person-years), 5921 T2D cases were observed. Compared to no noise exposure, the hazard ratios (HRs) and 95% confidence intervals (CIs) were 1.06 (0.95–1.18) in less than a year, 1.01 (0.91–1.12) in around 1–5 years, and 1.11 (1.04–1.20) in more than 5 years, respectively. Compared to participants with low genetic risk and no noise exposure, individuals with high genetic risk and noise exposure for more than 5 years did not show the highest risk of T2D (HR = 1.16, 95%CI = 0.98–1.36). However, participants with the least healthy lifestyle and noise exposure for more than 5 years revealed a higher risk of T2D (HR = 1.72, 95%CI = 1.27–2.33).

**Conclusions:**

Prolonged noise exposure in the workplace is related to a higher risk of T2D.

## 1. Introduction

Type 2 diabetes (T2D) has emerged as a major global public health issue over the past several decades [1]. According to the Global Burden of Diseases (GBD) 2021 [2, 3], T2D affected approximately 506 million individuals globally in 2021, accounting for 75 million disability-adjusted life years (DALYs). T2D is strongly associated with several clinical syndromes including diabetic foot ulcers [4], diabetic nephropathy [5], and diabetic cardiomyopathy [6]. Notably, the global distribution of the T2D burden has shifted over time: The country with the highest absolute T2D burden worldwide changed from China (in 1990) to India (by 2021). Additionally, the age-standardized DALY rate has increased by 0.42% per year from 1990 to 2021. Identifying risk factors of T2D and then controlling those might be an effective way for the prevention of T2D.

Environmental factors have been increasingly recognized as contributors to increasing the risk of T2D [7], with noise exposure emerging as a potentially impactful environmental determinant of T2D development [8]. For instance, previous epidemiological studies have indicated that traffic noise could increase the incidence of diabetes [9, 10] and diabetes mortality [11]. Notably, the majority of existing research on noise and T2D has focused exclusively on environmental noise. However, workplace noise exposure contrasts with environmental or social noise in three critical aspects: (1) sound pressure levels, (2) exposure duration, and (3) the spectral properties of the noise. [12]. These inherent differences suggest that the relationship between occupational noise and T2D may not be directly extrapolated from findings on environmental noise. A previous meta-analysis indicated that the overall evidence on the relationship between occupational noise exposure and diabetes is heterogeneous, limited, and mostly of low quality [13]. Thus, epidemiological studies with well-designed study design and large sample sizes are needed to evaluate the association between noise exposure in the workplace and T2D.

Studies have revealed that genetic risk and unhealthy lifestyle were both positively associated with the incidence of T2D [14, 15]. Studies have confirmed the interaction of environmental factors and genetic risk on T2D [16–18]. However, the influence of genetic susceptibility and lifestyles on the relationship between noise exposure and T2D is largely unknown.

Thus, we conducted this study with a large cohort from UK Biobank (UKB) to explore the association between noise exposure in the workplace and T2D. Furthermore, we assessed the impact of genetic susceptibility and lifestyle on the abovementioned relationship.

## 2. Research Design and Methods

### 2.1. Study Population

The UKB is a large-scale prospective cohort study that recruited 500,000 adults aged 40–69 years across the United Kingdom between 2006 and 2010. Information was collected via touchscreen questionnaires and structured interviews, standardized physical examinations, records of health outcomes, and high-quality biological samples such as blood and urine. A comprehensive description of study design, participant recruitment criteria, and data collection protocols has been published in previous literature [19].

The Application Number of our study was 88159. We excluded individuals with inaccurate information on noise exposure in the workplace (*n* = 332,109) and T2D at baseline (*n* = 10,035) and then excluded those with missing genetic data (*n* = 5549); finally, we included 154,708 participants for the relationship between noise exposure in the workplace and genetic susceptibility to T2D. With regard to lifestyle, we further excluded those with missing lifestyle data (*n* = 52,412), resulting in a total of 102,296 participants. The study flowchart is shown in Figure S1.

The ethics approval of the UKB study was granted by the North West Multi-Centre Research Ethics Committee. All enrolled individuals provided written informed consent in accordance with the principles of the Declaration of Helsinki.

### 2.2. Noise Exposure in the Workplace

Noise exposure in the workplace was assessed in the UKB using a touchscreen question. Participants were asked the following standardized question: “Have you ever worked in a noisy place where you had to shout to be heard?” (Field ID: 4825) [20]. This question was administered to all participants except for those who self-reported complete deafness, who were excluded from this specific assessment. The response options for the question were categorized into six mutually exclusive items, as follows: (1) yes, for more than 5 years; (2) yes, for around 1–5 years; (3) yes, for less than a year; (4) no; (5) do not know; and (6) prefer not to answer.

### 2.3. Identification of T2D

T2D was determined with the International Classification of Diseases-10th (ICD-10) revision code (E11); both primary and secondary diagnoses were included (Field ID: 41270).

### 2.4. Assessment of Genetic Risk

Genotype data were reported previously [21]. Genotyping was performed for all participants using either the UKB Axiom Array or the custom UK Biobank Lung Exome Variant Evaluation (UK BiLEVE) Axiom Array. For the present study, we utilized the standardized polygenic risk score (PRS) for T2D precomputed by the UKB. Participants were subsequently stratified into three genetic risk categories based on tertiles of this T2D PRS: low genetic risk (Tertile 1, < −0.58), medium genetic risk (Tertile 2, −0.58 < 0.23), and high genetic risk (Tertile 3, ≥ 0.23).

### 2.5. Lifestyle Score

In the present study, a lifestyle score was constructed to quantify overall lifestyle health, using eight modifiable lifestyle factors: sleep duration, physical activity, television viewing time, smoking status, alcohol intake, oily fish intake, vegetable intake and fruit, and red and processed meat intake [22]. For each of these eight factors, participants were assigned 1 point if they met the predefined “unhealthy” criterion (Table S1). Specifically, the “unhealthy” categories for each factor were defined as follows: current smoker; daily or almost daily alcohol intake; inadequate physical activity: < 150 min/week moderate or < 75 min/week vigorous physical activity; prolonged television viewing: ≥ 4 h/day of television viewing time; abnormal sleep duration: < 7 or > 9 h/day; insufficient fruit and vegetable intake: < 400 g/day; low oily fish intake: < 1 portion per week; and high red/processed meat intake: > 3 portions of red meat per week or > 1 portion of processed meat per week. An unweighted lifestyle score was calculated by summing the points from all eight factors. The lifestyle score ranged from 0 to 9, with a higher score indicating an unhealthier overall lifestyle. In addition, we also divided participants into three lifestyle groups to facilitate subsequent analyses: most healthy (0–2), moderately healthy (3–5), and least healthy (6–9) according to the lifestyle score [22].

### 2.6. Covariates

Basic information encompassing sociodemographic factors, physical measurements and medical history were collected via touchscreen questionnaires. Covariates incorporated in the analyses were selected following prior literature [23, 24], encompassing demographic factors (age, sex, country, and education), lifestyle variables (smoking status, alcohol consumption frequency, and hearing aid usage), anthropometric measures (body mass index (BMI)), socioeconomic indicators (Townsend deprivation index (tdi)), clinical conditions (hypertension and stroke), and environmental exposures (*L*_den_ and PM_2.5_). *L*_den_ representing the 24-h weighted traffic noise level, adjusts for evening and nighttime exposure by adding 5 and 10 dB penalties, respectively [25]. Comprehensive details on all covariates are provided in Table S2.

### 2.7. Statistical Analysis

Cox proportional hazards regression models were employed to evaluate the association of noise exposure in the workplace, genetic risk (based on T2D PRS) and lifestyle (based on the lifestyle score) with T2D. Results from these models were reported as hazard ratios (HRs) and 95% confidence intervals (CIs). Follow-up duration for each participant was calculated from the date of study recruitment to the earliest of the following time points: (1) date of first incidence of T2D, (2) date of death, (3) date of loss to follow-up, or (4) the end of follow-up (Dec 31, 2021), whichever came first [26]. Subgroup analyses were further performed stratified by sex and age to explore potential differences in associations across these subgroups. Additionally, sensitivity analyses were also conducted to evaluate the robustness of our primary findings. Initially, participants with incident T2D occurring within the first 3 years of follow-up were excluded to ensure disease onset after baseline. Subsequently, individuals with missing covariate data were omitted to validate the robustness of results through sensitivity analyses. Besides, the joint effect of noise exposure in the workplace and genetic risk with T2D, and the joint effect of noise exposure in the workplace and lifestyle with T2D were analyzed. Finally, we tested for potential interactions between noise exposure in the workplace (categorical variable) and PRS (categorical variable)/lifestyle (categorical variable).

Statistical analyses were performed using SAS software (ver. 9.4; SAS Institute Inc., Cary, NC, United States) and R software (Ver. 4.0.5) packages. Statistical tests were two-sided, and statistical significance was defined as a *p* value < 0.05.

### 2.8. STROBE Checklist Compliance

The study was conducted according to the completed STROBE checklist detailing compliance with all 22 items.

## 3. Results

Descriptive statistics of the enrolled cohort are summarized in Table 1. Among the 154,708 participants, 69,340 (44.8%) were male, with a mean age of 56.6 years. Individuals diagnosed with T2D exhibited statistically significant associations with a higher prevalence of smoking, alcohol consumption, hypertension, and stroke compared to non-T2D participants.

Table 2 shows the relationship between noise exposure in the workplace and the incidence of T2D. During a median follow-up of 11.83 years (1,776,919.62 person-years), 5921 T2D cases were observed. Compared to no noise exposure, the HRs and 95% CIs were 1.06 (0.95–1.18) in less than a year, 1.01 (0.91–1.12) in around 1–5 years, and 1.11 (1.04–1.20) in more than 5 years, respectively, after adjusting for potential confounders. In the subgroup analyses, the results were similar and the risk of T2D was higher in longer noise exposure in the workplace in almost all subgroups (Table S3). Meanwhile, these results were similar in the sensitivity analyses (Tables S4 and S5).

**Figure 1:**
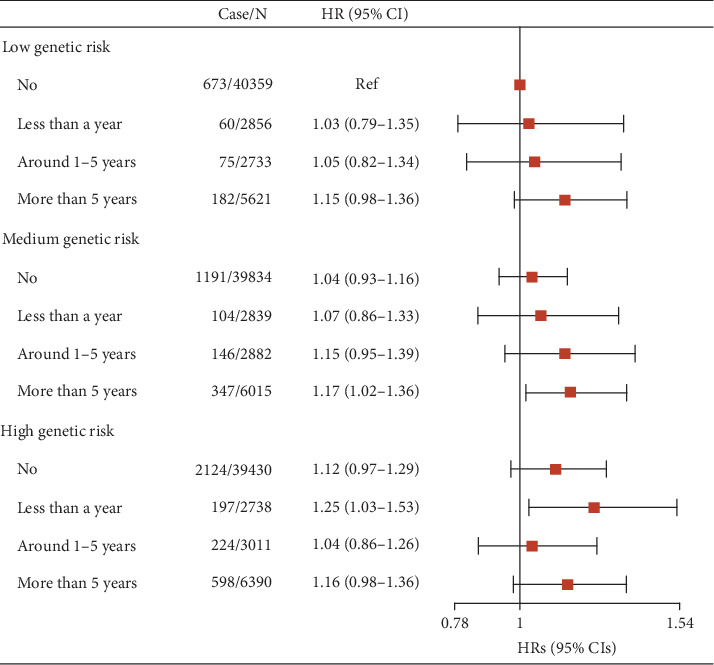
Joint association of noise exposure in the workplace and genetic risk with T2D (*N* = 154, 708). Adjusted for age, sex, country, education, smoking status, alcohol intake frequency, hearing aid use, BMI, tdi, hypertension, stroke, *L*_den_, and PM_2.5_.

The relationship between genetic risk and incident T2D is shown in Table S6. PRS was positively associated with T2D in the continuous model (HR = 1.63, 95%CI = 1.59–1.68). Compared to the low PRS category, the HR (95% CI) was 1.63 (1.50–1.76) for the medium genetic risk and 2.81 (2.61–3.02) for the high genetic risk, respectively. The positive association could also be found when further adjusting for PRS (HR = 1.08, 95%CI = 1.01–1.16) (Table S7).  Figure 1 shows the joint association of noise exposure in the workplace and genetic risk with T2D. Compared to no noise exposure and low genetic risk, those with high genetic risk and the longest noise exposure did not show the highest risk of T2D (HR = 1.16, 95%CI = 0.98–1.36). The interaction between noise exposure in the workplace and PRS was not statistically significant (*P*_interaction_ = 0.552) either.

**Figure 2:**
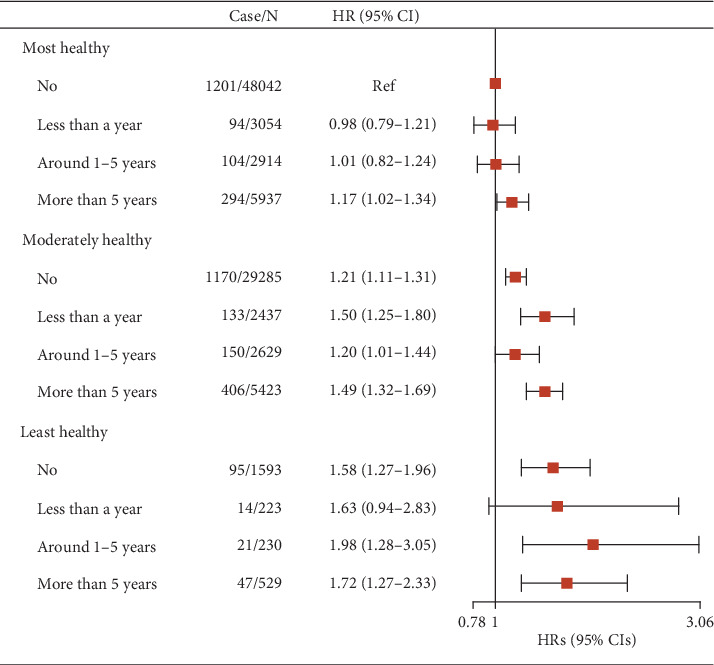
Joint association of noise exposure in the workplace and lifestyle score with T2D (*N* = 102,296). Adjusted for age, sex, country, education, hearing aid use, BMI, tdi, hypertension, stroke, *L*_den_, and PM_2.5_.

The associations of lifestyle categories and lifestyle score with risk of T2D are presented in Tables S8 and S9. The risk of T2D increased across lifestyle categories and scores. Lifestyle score was positively associated with T2D in the continuous model (HR = 1.11, 95%CI = 1.09–1.14). Compared to the most healthy category, the HR (95% CI) was 1.24 (1.16–1.33) for the moderately healthy and 1.59 (1.36–1.87) for the least healthy, respectively. The joint association of noise exposure in the workplace and lifestyle with T2D is presented in Figure 2. Compared to no noise exposure and the most healthy lifestyle, those with the least healthy lifestyle and the longest noise exposure showed a higher risk of T2D (HR = 1.72, 95%CI = 1.27–2.33). However, the interaction between noise exposure in the workplace and lifestyle was not statistically significant (*P*_interaction_ = 0.601). For the specific lifestyle (Table S10), the positive association could be found in almost all the subgroups.

## 4. Discussion

The study found that noise exposure lasting more than 5 years in the workplace was associated with a higher risk of incident T2D based on a large prospective cohort study. Concurrently, both genetic risk and unhealthy lifestyle were independently positively related to a higher risk of incident T2D. While the statistical test for interaction was not significant, the stratified analysis revealed evidence of a joint association between noise exposure and lifestyle on T2D. Of course, it does not imply a synergistic interaction in the statistical sense.

Noise exposure is common in the workplace worldwide [27]. Our previous studies have found that occupational noise exposure was related to early cardiovascular injury [28–30]. The present study verified the positive association between occupational noise exposure and T2D, which indicated that noise control in the workplace is necessary for the prevention of T2D. A recent meta-analysis indicated that the positive association between occupational noise and diabetes was not statistically significant [13], including combined cohort studies (RR = 1.17, 95%CI = 0.84–1.50) and cross-sectional studies (OR = 1.26, 95%CI = 0.93–1.58). However, the meta-analysis also indicated that the present studies are heterogeneous and mostly of low quality. Only three cohort studies were included in the meta-analysis, and our result was similar to a cohort study included in the meta-analysis with a large sample size [31]. However, the cohort study just assessed the relationship between occupational noise and gestational diabetes among female workers. Our study assessed the relationship between noise exposure in the workplace and T2D with a large sample size and longer follow-up time, and we also adjusted for a comprehensive set of potential confounders, including other prominent environmental factors (traffic noise and PM_2.5_) that have been previously linked to T2D risk. Thus, we hold that the present study could provide a more accurate result on the topic between occupational noise and T2D.

Actually, the observed HR of noise exposure in the workplace for more than 5 years for T2D represents a modest effect size though statistically significant. Compared with other established risk factors, such as shift work [32], genetic risk, and an unhealthy lifestyle, the effect of noise exposure in the workplace on T2D may be relatively smaller. Given that occupational noise exposure is highly prevalent globally, even a modest increase in individual T2D risk can translate to a substantial number of excess T2D cases at the population level, underscoring its nonnegligible public health implications despite the small effect size. In the subgroup analyses of specific lifestyles, a smaller number may result in limited statistical power and wider CIs, including smoking, fruit and vegetable intake, and oily fish intake.

In addition, the joint relationship between noise exposure in the workplace and lifestyle with T2D was statistically significant, but not for genetic risk. The results indicated that the elevated T2D risk associated with long-term noise exposure in the workplace could be further amplified by an unhealthy lifestyle, but not by high genetic risk. This pattern could be explained by the fact that lifestyle plays a more important role in the occurrence of T2D among noise-exposed workers. A recent study quantified the relative contributions of environment and genetics to aging and premature mortality [33] and found that the environment explained a greater proportion of variation compared with genetic risk for the incidence of several diseases, which may support our findings. Importantly, lifestyle factors are modifiable, which highlights a critical opportunity for intervention. Thus, implementing targeted measures for the prevention and control of noise exposure in the workplace and advocating for the adoption of a healthy lifestyle are both needed to reduce the risk of T2D among noise-exposed workers.

The mechanism between noise exposure in the workplace and T2D remains incompletely understood. It is reported that noise could stimulate the arousal/sympathetic nervous system and the hypothalamic–pituitary–adrenal axis, then increasing the level of related hormones, including cortisol, adrenaline, and noradrenaline [34, 35], which may be related to the development of T2D. Meanwhile, noise exposure may directly alter carbohydrate metabolism by activating signaling pathways, such as the unfolded protein response and stress-activated mitogen-activated protein kinase (MAPK) pathways [36], which then change insulin signaling, insulin activity, immune responses, and blood glucose levels. Besides, our previous study indicated that environmental stimuli including occupational noise exposure could affect obesity-related indices and lipid metabolism; those factors are also risk factors of T2D [37–40]. These factors—obesity and dyslipidemia—are well-established independent risk factors for T2D, suggesting that they may act as intermediate mediators linking occupational noise exposure to T2D risk. Furthermore, an unhealthy lifestyle could also affect the abovementioned mechanism, resulting in a joint relationship between noise exposure in the workplace and unhealthy lifestyle on T2D risk. For instance, an unhealthy lifestyle was reported to be involved in the stress response, including the hypothalamic–pituitary–adrenal axis, inflammation and the autonomic nervous system, and then impact biological systems [41]. Meanwhile, an unhealthy lifestyle also directly disrupts carbohydrate metabolism (e.g., by impairing insulin signaling) [42] and lipid metabolism [43], further augmenting T2D risk. Thus, noise exposure and an unhealthy lifestyle may simultaneously affect the hypothalamic–pituitary–adrenal axis; inflammation markers and cytokines, such as tumor necrosis factor-alpha (TNF-*α*) and interleukin-1 (IL-1), interleukin-6 (IL-6), and interleukin-8 (IL-8); and carbohydrate metabolism like stress-activated MAP kinase pathways, and so forth.

The present study has several advantages. Our study was conducted with a larger sample size and longer follow-up time. Furthermore, environmental risk factors (traffic noise and PM_2.5_) were also adjusted when evaluating the relationship between noise exposure in the workplace and T2D, making the results more reliable. Finally, the assessment of the joint relationship of noise exposure in the workplace and genetic/lifestyle with the risk of T2D provides novel insights into the combined influence of these exposures on T2D incidence. Limitations should also be noted. First, noise exposure was self-reported and assessed using noise exposure time, though our previous studies have indicated that noise exposure time could also provide related reliable results [44, 45]. This subjective assessment might not reflect the actual individual noise level compared with studies using objective measures [29, 46]; it might lead to nondifferential misclassification, which would typically bias the results towards the null, and influence the magnitude and precision of the reported HRs. Finally, it limited the ability to establish a precise dose–response relationship based on actual sound levels, which could not translate findings into specific quantitative occupational health guidelines. Thus, we hold that noise exposure in the workplace should be assessed with cumulative noise exposure combining noise exposure level (dB(A)) and duration of exposed noise in future studies. Second, selection bias may affect the results by excluding 52,412 participants with missing lifestyle data, as some differences in the baseline characteristics were found between the 102,296 participants included and the 52,412 excluded from lifestyle analyses (Table S11). Sensitivity analyses excluding missing covariates could not fully address this problem. Third, the generalizability of findings to global or ethnically diverse populations may be limited, as the study cohort was predominantly recruited from England. Additionally, the exclusion of participants with incomplete lifestyle data introduces potential selection bias. Fourth, the assessment of noise exposure and lifestyle factors at a single baseline time point may not capture changes over the follow-up period. Finally, some other residual confounders were not considered, particularly from unmeasured occupational factors associated with both noise exposure and T2D risk; for instance, coexposure to occupational chemicals or specific work-related stress patterns not captured by the general lifestyle score. Therefore, future studies should focus more on objective noise measurement protocols, longitudinal assessment of noise exposure and lifestyle factors; meanwhile, future research should also aim to disentangle the effects of different noise characteristics (e.g., frequency and intermittency) and investigate the biological mechanisms (e.g., via repeated biomarker measurements) linking noise, lifestyle, and T2D development.

## 5. Conclusions

Our analysis demonstrated a statistically significant positive association between long-term occupational noise exposure and elevated risk of T2D. Notably, a joint association is found for noise exposure in the workplace and lifestyle on T2D, but not for genetic risk. These findings underscore the importance of implementing noise control strategies in occupational settings, such as engineering controls and policy interventions, to mitigate T2D risk among workers exposed to noise environments.

## Figures and Tables

**Table 1:** Baseline characteristics of participants included in the study (*N* = 154,708).

**Characteristics,** **n** ** (%)**	**Total (** **n** = 154,708**)**	**With Type 2 diabetes (** **n** = 5921**)**	**Without Type 2 diabetes (** **n** = 148,787**)**	**p** ** values**
Age, year	56.6 ± 8.2	59.1 ± 7.4	56.5 ± 8.2	< 0.001
Male	69,340 (44.8)	3317 (56.0)	66,023 (44.4)	< 0.001
Country				< 0.001
England	152,242 (98.4)	5888 (99.4)	146,354 (98.4)	
Wales	2466 (1.6)	33 (0.6)	2433 (1.6)	
Education				< 0.001
Higher	52,770 (34.1)	1274 (21.5)	51,496 (34.6)	
Upper secondary	50,801 (32.8)	1689 (28.5)	49,112 (33.0)	
Lower secondary	8823 (5.7)	363 (6.1)	8460 (5.7)	
Vocational	18,075 (11.7)	882 (14.9)	17,193 (11.6)	
No secondary education	22,698 (14.7)	1597 (27.0)	21,101 (14.2)	
Prefer not to answer	1541 (1.0)	116 (2.0)	1425 (1.0)	
Smoking status				< 0.001
Never	85,870 (55.5)	2820 (47.6)	83,050 (55.8)	
Previous	52,857 (34.2)	2260 (38.2)	50,597 (34.0)	
Current	15,450 (10.0)	803 (13.6)	14,647 (9.8)	
Prefer not to answer	531 (0.3)	38 (0.6)	493 (0.3)	
Alcohol intake frequency				< 0.001
Daily or almost daily	31,998 (20.7)	871 (14.7)	31,127 (20.9)	
3–4 times/week	35,176 (22.7)	987 (16.7)	34,189 (23.0)	
1–2 times/week	39,090 (25.3)	1345 (22.7)	37,745 (25.4)	
1–3 times/month	17,500 (11.3)	724 (12.2)	16,776 (11.3)	
Special occasions only	18,136 (11.7)	1092 (18.4)	17,044 (11.5)	
Never	12,682 (8.2)	892 (15.1)	11,790 (7.9)	
Prefer not to answer	126 (0.1)	10 (0.2)	116 (0.1)	
Hearing aid use	4418 (2.9)	316 (5.3)	4102 (2.8)	< 0.001
BMI	27.2 ± 4.6	31.2 ± 5.5	27.0 ± 4.5	< 0.001
Townsend deprivation index	−1.17 ± 2.92	−0.29 ± 3.21	−1.21 ± 2.90	< 0.001
PM_2.5_	9.9 ± 0.9	10.0 ± 0.9	9.9 ± 0.9	< 0.001
*L*_den_	55.9 ± 4.2	56.0 ± 4.3	55.9 ± 4.2	0.002
Hypertension	39,501 (25.5)	2888 (48.8)	36,613 (24.6)	< 0.001
Stroke	2029 (1.3)	208 (3.5)	1821 (1.2)	< 0.001

*Note:* Data are mean ± SD or frequencies (percentages).

Abbreviations: BMI, body mass index; *L*_den_: 24-h traffic noise;; PM_2.5_, fine particulate matter with diameter < 2.5* μ*m SD: standard deviation; tdi, Townsend deprivation index.

**Table 2:** Association between noise exposure in the workplace and risk of Type 2 diabetes (*N* = 154,708).

**Noise exposure in the workplace**	**Case/** **N**	**HR (95% CI)**		
		**Model 1**	**Model 2**	**Model 3**
No	3988/119,623	Ref	Ref	Ref
Less than a year	361/8433	1.29 (1.16–1.44)	1.19 (1.07–1.33)	1.06 (0.95–1.18)
Around 1–5 years	445/8626	1.57 (1.43–1.74)	1.43 (1.30–1.58)	1.01 (0.91–1.12)
More than 5 years	1127/18,026	1.94 (1.81–2.07)	1.58 (1.48–1.70)	1.11 (1.04–1.20)

*Note:* Model 1: unadjusted; Model 2: adjusted for age and sex; Model 3: adjusted for age, sex, country, education, smoking status, alcohol intake frequency, hearing aid use, BMI, tdi, hypertension, stroke, *L*_den_, and PM_2.5_.

Abbreviations: BMI, body mass index; CI, confidence interval; HRs, hazard ratios; *L*_den_: 24-h traffic noise; PM_2.5_, fine particulate matter with diameter < 2.5* μ*m; tdi, Townsend deprivation index.

## Data Availability

Data could be available on reasonable request with the corresponding author.

## Supporting Information

Supporting Information Additional supporting information can be found online in the Supporting Information section. Table S1: Variables used to create lifestyle score for UK Biobank. Table S2: Covariates definitions and assessment in our study. Table S3: Subgroup analyses for association between noise exposure in the workplace and risk of Type 2 diabetes (*N* = 154,708). Table S4: Sensitivity analyses for association between noise exposure in the workplace and risk of Type 2 diabetes excluding Type 2 diabetes within 3 years (*N* = 152,842). Table S5: Sensitivity analyses for association between noise exposure in the workplace and risk of Type 2 diabetes excluding missing covariates (*N* = 151,676). Table S6: Association between genetic risk and incident Type 2 diabetes (*N* = 154,708). Table S7: Association between noise exposure in the workplace and risk of Type 2 diabetes adjusted for PRS (*N* = 154, 708). Table S8: Association between lifestyle category and risk of Type 2 diabetes (*N* = 102,296). Table S9: Association between lifestyle score and risk of Type 2 diabetes (*N* = 102,296). Table S10: Association between lifestyle factors and risk of Type 2 diabetes (*N* = 102,296). Table S11: Characteristics of participants with (*N* = 102,296) and without (*N* = 52,412) lifestyle data. Figure S1: Flowchart of the study.
