# Supplementary material for: Association of Noise Exposure, Genetic Susceptibility, and Lifestyle With Type 2 Diabetes: A Prospective Cohort Study
Source: J Diabetes Res. 2025 Nov 18;2025:8974749. doi: 10.1155/jdr/8974749 (PMC12646724; doi:10.1155/jdr/8974749)
Supplement: Supporting Information — Additional supporting information can be found online in the Supporting Information section. Table S1: Variables used to create lifestyle score for UK Biobank. Table S2: Covariates definitions and assessment in our study. Table S3: Subgroup analyses for association between noise exposure in the workplace and risk of Type 2 diabetes (N = 154,708). Table S4: Sensitivity analyses for association between noise exposure in the workplace and risk of Type 2 diabetes excluding Type 2 diabetes within 3 years (N = 152,842). Table S5: Sensitivity analyses for association between noise exposure in the workplace and risk of Type 2 diabetes excluding missing covariates (N = 151,676). Table S6: Association between genetic risk and incident Type 2 diabetes (N = 154,708). Table S7: Association between noise exposure in the workplace and risk of Type 2 diabetes adjusted for PRS (N = 154, 708). Table S8: Association between lifestyle category and risk of Type 2 diabetes (N = 102,296). Table S9: Association between lifestyle score and risk of Type 2 diabetes (N = 102,296). Table S10: Association between lifestyle factors and risk of Type 2 diabetes (N = 102,296). Table S11: Characteristics of participants with (N = 102,296) and without (N = 52,412) lifestyle data. Figure S1: Flowchart of the study. [file 8974749.f1.docx]

**Table S1 Variables used to create lifestyle score for UK Biobank**

| **Lifestyle factors** | **Questionnaire** | **‘Healthy’** | **‘Unhealthy’** |
| --- | --- | --- | --- |
| Smoking status | "Do you smoke tobacco now?" and "In the past, how often have you smoked tobacco?" | Past or never smoker | Current |
| Alcohol intake | “About how often do you drink alcohol?” | ≤ 4 times week | Daily or almost daily |
| Physical activity | IPAQ short form2– total time walking or moderate and vigorous-intensity physical activity (PA) in previous week | ≥150 min/week moderate or ≥ 75 min/week vigorous PA | <150 min/week moderate or < 75 min/week vigorous PA |
| TV viewing time | "In a typical day, how many hours do you spend watching TV?" | < 4 h/day | ≥ 4 h/day |
| Sleep time | “About how many hours sleep do you get in every 24 hours?” | >7 or <9h/day | <7 or >9h/day |
| Dietary characteristics | Individual dietary components contributed directly to lifestyle score. |  |  |
| Fruit and vegetable intake | "About how many of …. would you eat per day?” Separate questions for pieces of fresh and dried fruit, tablespoons of salad or cooked/raw vegetables. Combined and converted to g/day (1 portion = 80 g) | ≥ 400 g/day | <400 g/ day |
| Oily fish intake | "How often do you eat oily fish? (e.g. sardines, salmon, mackerel, herring)" | ≥1 portion/week | <1 portion/week |
| Red meat intake | "How often do you eat…?” Separate questions for Beef / lamb or mutton / pork (excluding processed meats such as ham or bacon). Red meat included due to clear link between red meat and mortality. | ≤3 portion/week | >3 portion/week |
| Processed meat intake | "How often do you eat processed meats (such as bacon, ham, sausages, meat pies, kebabs, burgers, chicken nuggets)?" | ≤1 portion/week | >1 portion/week |

Participants were classified to healthy or unhealthy categories based on their responses to questions for each lifestyle factor. UK Biobank physical activity (PA) data were analyzed in accordance with the International Physical Activity Questionnaire (IPAQ) scoring protocol with total physical activity computed as the sum of walking, moderate and vigorous activity, measured as metabolic equivalents (MET-hours/week). National dietary guidelines were used as the basis for the dietary components. UK Biobank dietary information was collected via the Oxford WebQ; a web-based 24-hour recall questionnaire developed specifically for use in large population studies.

**Table S2 Covariates definitions and assessment in our study**

| **Covariates** | **Description** | **Assessment** | **UK Biobank Code** |
| --- | --- | --- | --- |
| Age (year) | Age when attended assessment center | Based on date of birth and date of attending assessment center | 21003 |
| Sex | Male, female | NHS had recorded for the participant and self-reported sex | 31 |
| Country | England, Wales | The UK Biobank assessment center at which participant consented | 54 |
| Education | Higher (College or University degree), Upper secondary (A levels/AS levels or equivalent, O levels/GCSEs or equivalent), Lower secondary (CSEs or equivalent), Vocational (NVQ or HND or HNC or equivalent, Other professional qualifications), No secondary education (None of the above) | Touchscreen question "Which of the following qualifications do you have? | 6138 |
| Smoking status | Never, previous, current | Touchscreen question | 21006 |
| Alcohol intake frequency | Daily or almost daily, 3–4 times/week, 1–2 times/week, 1–3 times/month, special occasions only, never | Touchscreen question "About how often do you drink alcohol?" | 1558 |
| Hearing aid use | Yes, no | Touchscreen question "Do you use a hearing aid most of the time?" | 3393 |
| Hypertension | Yes, no | Touchscreen questionnaire and verbal interview: self-reported hypertension or anti-hypertensive medication use | 6150, 6177, 20002 |
| Diabetes | Yes, no | Touchscreen questionnaire and verbal interview: self-reported diabetes (diabetes, type 1 diabetes or type 2 diabetes) or insulin use | 2443, 6177, 20002 |
| Stroke | Yes, no | Touchscreen questionnaire and verbal interview: self-reported previous stroke | 6150, 20002 |
| BMI | Body mass index | Constructed from height and weight measured during the initial Assessment Centre visit. | 21001 |
| Townsend deprivation index | Continuous | Townsend deprivation index calculated immediately prior to participant joining UK Biobank. | 189 |
| PM_2.5_ | Continuous | Particulate matter air pollution (pm2.5); 2010 | 24006 |
| L_den_ | Continuous | Weighted average 24-h noise sound level, with a penalty of 5 and 10 dB added to the evening hours and night hours, respectively | 24020, 24021, 24022 |

Abbreviations: BMI, body mass index; L_den_: 24-h traffic noise; PM_2.5_, fine particulate matter with diameter < 2.5 μm.

**Table S3 Subgroup analyses for association between noise exposure in the workplace and risk of type 2 diabetes (N=154,708)**

| **Noise exposure in the workplace** | **Case/N** | **HR (95% CI)** | | |
| --- | --- | --- | --- | --- |
|  |  | **Model 1** | **Model 2** | **Model 3** |
| **Male** |  |  |  |  |
| No | 1,833/43,658 | Ref | Ref | Ref |
| Less than a year | 283/6,188 | 1.09 (0.96-1.24) | 1.12 (0.99-1.41) | 1.04 (0.91-1.18) |
| Around 1-5 years | 297/5,757 | 1.24 (1.09-1.40) | 1.25 (1.11-1.41) | 0.92 (0.81-1.05) |
| More than 5 years | 904/13,737 | 1.61 (1.49-1.74) | 1.51 (1.40-1.64) | 1.07 (0.98-1.17) |
| **Female** |  |  |  |  |
| No | 2,155/75,965 | Ref | Ref | Ref |
| Less than a year | 78/2,245 | 1.22 (0.98-1.53) | 1.35 (1.07-1.69) | 1.05 (0.84-1.33) |
| Around 1-5 years | 148/2,869 | 1.85 (1.57-2.19) | 1.89 (1.60-2.23) | 1.20 (1.01-1.43) |
| More than 5 years | 223/4,289 | 1.86 (1.62-2.14) | 1.76 (1.53-2.02) | 1.21 (1.05-1.40) |
| **Age <60** |  |  |  |  |
| No | 1,714/67,161 | Ref | Ref | Ref |
| Less than a year | 183/5,304 | 1.36 (1.17-1.58) | 1.19 (1.02-1.39) | 1.06 (0.91-1.24) |
| Around 1-5 years | 213/4,893 | 1.73 (1.50-1.99) | 1.54 (1.33-1.77) | 1.05 (0.90-1.21) |
| More than 5 years | 409/8,373 | 1.95 (1.75-2.17) | 1.67 (1.50-1.87) | 1.16 (1.03-1.30) |
| **Age ≥60** |  |  |  |  |
| No | 2,274/52,462 | Ref | Ref | Ref |
| Less than a year | 178/3,129 | 1.33 (1.14-1.55) | 1.18 (1.01-1.37) | 1.03 (0.88-1.21) |
| Around 1-5 years | 232/3,733 | 1.47 (1.28-1.68) | 1.34 (1.16-1.53) | 0.98 (0.85-1.12) |
| More than 5 years | 718/9,653 | 1.78 (1.64-1.94) | 1.58 (1.45-1.73) | 1.11 (1.01-1.22) |

Model 1: unadjusted;

Model 2: adjusted for age/sex;

Model 3: adjusted for age/sex, country, education, smoking status, alcohol intake frequency, hearing aid use, BMI, tdi, hypertension, stroke, L_den_, PM_2.5_.

Abbreviations: BMI, body mass index; HR, hazard ratio; CI, confidential interval; tdi, townsend deprivation index, L_den_: 24-h traffic noise; PM_2.5_, fine particulate matter with diameter < 2.5 μm.

**Table S4 Sensitivity analyses for association between noise exposure in the workplace and risk of type 2 diabetes excluding type 2 diabetes within three years (N=152,842)**

| **Noise exposure in the workplace** | **Case/N** | **HR (95% CI)** | | |
| --- | --- | --- | --- | --- |
|  |  | **Model 1** | **Model 2** | **Model 3** |
| No | 3,548/118,361 | Ref | Ref | Ref |
| Less than a year | 317/8,317 | 1.28 (1.14-1.43) | 1.18 (1.05-1.33) | 1.06 (0.94-1.19) |
| Around 1-5 years | 395/8,489 | 1.57 (1.42-1.75) | 1.44 (1.29-1.60) | 1.01 (0.91-1.13) |
| More than 5 years | 996/17,675 | 1.93 (1.80-2.07) | 1.59 (1.48-1.71) | 1.11 (1.03-1.20) |

Model 1: unadjusted;

Model 2: adjusted for age, sex;

Model 3: adjusted for age, sex, country, education, smoking status, alcohol intake frequency, hearing aid use, BMI, tdi, hypertension, stroke, L_den_, PM_2.5_.

Abbreviations: BMI, body mass index; HR, hazard ratio; CI, confidential interval; tdi, townsend deprivation index, L_den_: 24-h traffic noise; PM_2.5_, fine particulate matter with diameter < 2.5 μm.

**Table S5 Sensitivity analyses for association between noise exposure in the workplace and risk of type 2 diabetes excluding missing covariates (N=151,676)**

| **Noise exposure in the workplace** | **Case/N** | **HR (95% CI)** | | |
| --- | --- | --- | --- | --- |
|  |  | **Model 1** | **Model 2** | **Model 3** |
| No | 3,877/117,271 | Ref | Ref | Ref |
| Less than a year | 351/8,248 | 1.30 (1.16-1.45) | 1.19 (1.07-1.33) | 1.06 (0.95-1.18) |
| Around 1-5 years | 429/8,443 | 1.57 (1.42-1.73) | 1.42 (1.28-1.57) | 1.01 (0.91-1.12) |
| More than 5 years | 1,104/17,714 | 1.95 (1.82-2.08) | 1.59 (1.48-1.71) | 1.11 (1.04-1.20) |

Model 1: unadjusted;

Model 2: adjusted for age, sex;

Model 3: adjusted for age, sex, country, education, smoking status, alcohol intake frequency, hearing aid use, BMI, tdi, hypertension, stroke, L_den_, PM_2.5_.

Abbreviations: BMI, body mass index; HR, hazard ratio; CI, confidential interval; tdi, townsend deprivation index, L_den_: 24-h traffic noise; PM_2.5_, fine particulate matter with diameter < 2.5 μm.

**Table S6 Association between genetic risk and incident type 2 diabetes (N=154,708)**

|  | **N** | **Case** | **HR (95% CI)** |
| --- | --- | --- | --- |
| Continuous | 154,708 | 5,921 | 1.63 (1.59-1.68) |
| Categories of PRS according to tertile | | |  |
| Low (tertile 1) | 51,569 | 990 | Ref |
| Medium (tertile 2) | 51,570 | 1,788 | 1.63 (1.50-1.76) |
| High (tertile 3) | 51,569 | 3,143 | 2.81 (2.61-3.02) |

Adjusted for age, sex, country, education, smoking status, alcohol intake frequency, hearing aid use, BMI, tdi, hypertension, stroke, L_den_, PM_2.5_.

Abbreviations: BMI, body mass index; HR, hazard ratio; CI, confidential interval; tdi, townsend deprivation index, L_den_: 24-h traffic noise; PM_2.5_, fine particulate matter with diameter < 2.5 μm.

**Table S7 Association between noise exposure in the workplace and risk of type 2 diabetes adjusted for PRS (N=154,708)**

| **Noise exposure in the workplace** | **Case/N** | **HR (95% CI)** |
| --- | --- | --- |
| No | 3,988/119,623 | Ref |
| Less than a year | 361/8,433 | 1.07 (0.96-1.20) |
| Around 1-5 years | 445/8,626 | 1.00 (0.90-1.11) |
| More than 5 years | 1,127/18,026 | 1.08 (1.01-1.16) |

Model 1: unadjusted;

Model 2: adjusted for age, sex;

Model 3: adjusted for age, sex, country, smoking status, alcohol intake frequency, education, hearing aid use, BMI, tdi, hypertension, stroke, L_den_, PM_2.5_, PRS.

Abbreviations: BMI, body mass index; HR, hazard ratio; CI, confidential interval; tdi, townsend deprivation index, L_den_: 24-h traffic noise; PM_2.5_, fine particulate matter with diameter < 2.5 μm. PRS: polygenic risk score.

**Table S8 Association between lifestyle category and risk of type 2 diabetes (N=102,296)**

|  | **N** | **Case** | **HR (95% CI)** |
| --- | --- | --- | --- |
| Continuous | 102296 | 3729 | 1.11 (1.09-1.14) |
| Categories of lifestyle score | | |  |
| Most healthy (0-2) | 59947 | 1693 | Ref |
| Moderately healthy (3-5) | 39774 | 1859 | 1.24 (1.16-1.33) |
| Least healthy (6-9) | 2575 | 177 | 1.59 (1.36-1.87) |

Adjusted for age, sex, country, education, hearing aid use, BMI, tdi, hypertension, stroke, L_den_, PM_2.5_.

Abbreviations: BMI, body mass index; HR, hazard ratio; CI, confidential interval; tdi, townsend deprivation index, L_den_: 24-h traffic noise; PM_2.5_, fine particulate matter with diameter < 2.5 μm.

**Table S9 Association between lifestyle score and risk of type 2 diabetes (N=102,296)**

| **Lifestyle score** | **N** | **Case** | **HR (95% CI)** |
| --- | --- | --- | --- |
| 0 (Most healthy) | 9708 | 175 | Ref |
| 1 | 22877 | 548 | 1.15 (0.97-1.37) |
| 2 | 27362 | 970 | 1.49 (1.27-1.81) |
| 3 | 21419 | 916 | 1.58 (1.34-1.86) |
| 4 | 12676 | 586 | 1.53 (1.28-1.81) |
| 5 | 5679 | 357 | 1.96 (1.63-2.36) |
| 6 | 1934 | 139 | 2.16 (1.72-2.71) |
| ≥7 (Least healthy) | 641 | 38 | 1.91 (1.34-2.74) |

Adjusted for age, sex, country, education, hearing aid use, BMI, tdi, hypertension, stroke, L_den_, PM_2.5_.

Abbreviations: BMI, body mass index; HR, hazard ratio; CI, confidential interval; tdi, townsend deprivation index, L_den_: 24-h traffic noise; PM_2.5_, fine particulate matter with diameter < 2.5 μm.

**Table S10 Association between lifestyle factors and risk of type 2 diabetes (N=102,296)**

| **Lifestyle factors** | **N** | **Case** | **HR (95% CI)** |
| --- | --- | --- | --- |
| Smoking status (healthy) |  |  |  |
| No | 72362 | 2188 | Ref |
| Less than a year | 4998 | 212 | 1.16 (1.00-1.34) |
| Around 1-5 years | 4928 | 226 | 1.01 (0.88-1.16) |
| More than 5 years | 10351 | 635 | 1.21 (1.10-1.34) |
| Smoking status (unhealthy) |  |  |  |
| No | 6558 | 278 | Ref |
| Less than a year | 716 | 29 | 0.86 (0.58-1.27) |
| Around 1-5 years | 845 | 49 | 1.01 (0.73-1.38) |
| More than 5 years | 1538 | 112 | 1.18 (0.93-1.50) |
| Alcohol intake (healthy) |  |  |  |
| No | 62153 | 2086 | Ref |
| Less than a year | 4327 | 201 | 1.13 (0.98-1.32) |
| Around 1-5 years | 4657 | 228 | 0.98 (0.85-1.12) |
| More than 5 years | 9458 | 613 | 1.17 (1.06-1.29) |
| Alcohol intake (unhealthy) |  |  |  |
| No | 16767 | 380 | Ref |
| Less than a year | 1387 | 40 | 0.99 (0.71-1.37) |
| Around 1-5 years | 1116 | 47 | 1.14 (0.83-1.55) |
| More than 5 years | 2431 | 134 | 1.25 (1.01-1.55) |
| Physical activity (healthy) |  |  |  |
| No | 42559 | 1005 | Ref |
| Less than a year | 3461 | 115 | 1.08 (0.89-1.32) |
| Around 1-5 years | 3322 | 123 | 1.01 (0.83-1.22) |
| More than 5 years | 7007 | 343 | 1.20 (1.05-1.38) |
| Physical activity (unhealthy) |  |  |  |
| No | 36361 | 1461 | Ref |
| Less than a year | 2253 | 126 | 1.18 (0.98-1.42) |
| Around 1-5 years | 2451 | 152 | 1.02 (0.86-1.21) |
| More than 5 years | 4882 | 404 | 1.23 (1.09-1.38) |
| TV viewing time (healthy) |  |  |  |
| No | 58211 | 1527 | Ref |
| Less than a year | 4177 | 149 | 1.06 (0.89-1.26) |
| Around 1-5 years | 3758 | 137 | 0.92 (0.77-1.10) |
| More than 5 years | 7197 | 377 | 1.15 (1.02-1.30) |
| TV viewing time (unhealthy) |  |  |  |
| No | 20709 | 939 | Ref |
| Less than a year | 1537 | 92 | 1.16 (0.93-1.45) |
| Around 1-5 years | 2015 | 138 | 1.10 (0.92-1.33) |
| More than 5 years | 4692 | 370 | 1.26 (1.10-1.44) |
| Sleep duration (healthy) |  |  |  |
| No | 58975 | 1637 | Ref |
| Less than a year | 4106 | 157 | 1.09 (0.92-1.29) |
| Around 1-5 years | 4002 | 167 | 0.97 (0.83-1.15) |
| More than 5 years | 8376 | 485 | 1.17 (1.04-1.30) |
| Sleep duration (unhealthy) |  |  |  |
| No | 19945 | 829 | Ref |
| Less than a year | 1608 | 84 | 1.11 (0.88-1.40) |
| Around 1-5 years | 1771 | 108 | 1.04 (0.84-1.28) |
| More than 5 years | 3513 | 262 | 1.25 (1.07-1.46) |
| Fruit and vegetable intake (healthy) |  |  |  |
| No | 67361 | 2009 | Ref |
| Less than a year | 4618 | 182 | 1.07 (0.91-1.25) |
| Around 1-5 years | 4643 | 218 | 1.04 (0.90-1.20) |
| More than 5 years | 9672 | 611 | 1.27 (1.15-1.40) |
| Fruit and vegetable intake (unhealthy) |  |  |  |
| No | 11559 | 457 | Ref |
| Less than a year | 1096 | 59 | 1.25 (0.95-1.65) |
| Around 1-5 years | 1130 | 57 | 0.89 (0.67-1.18) |
| More than 5 years | 2217 | 136 | 0.96 (0.78-1.19) |
| Oily fish intake (healthy) |  |  |  |
| No | 46032 | 1349 | Ref |
| Less than a year | 3126 | 124 | 1.11 (0.92-1.34) |
| Around 1-5 years | 3185 | 146 | 1.03 (0.87-1.24) |
| More than 5 years | 6683 | 426 | 1.27 (1.13-1.44) |
| Oily fish intake (unhealthy) |  |  |  |
| No | 32888 | 1117 | Ref |
| Less than a year | 2588 | 117 | 1.11 (0.91-1.35) |
| Around 1-5 years | 2588 | 129 | 0.97 (0.81-1.18) |
| More than 5 years | 5206 | 321 | 1.11 (0.97-1.27) |
| Red meat intake (healthy) |  |  |  |
| No | 69266 | 2095 | Ref |
| Less than a year | 4842 | 191 | 1.08 (0.93-1.26) |
| Around 1-5 years | 4836 | 218 | 0.99 (0.85-1.14) |
| More than 5 years | 9904 | 594 | 1.18 (1.07-1.31) |
| Red meat intake (unhealthy) |  |  |  |
| No | 9654 | 371 | Ref |
| Less than a year | 872 | 50 | 1.18 (0.87-1.60) |
| Around 1-5 years | 937 | 57 | 1.05 (0.78-1.41) |
| More than 5 years | 1985 | 153 | 1.26 (1.02-1.56) |
| Processed meat intake (healthy) |  |  |  |
| No | 57035 | 3140 | Ref |
| Less than a year | 3413 | 208 | 0.99 (0.82-1.20) |
| Around 1-5 years | 3506 | 258 | 0.87 (0.72-1.04) |
| More than 5 years | 7002 | 489 | 1.17 (1.04-1.32) |
| Processed meat intake (unhealthy) |  |  |  |
| No | 1126 | 21885 | Ref |
| Less than a year | 2301 | 124 | 1.26 (1.03-1.54) |
| Around 1-5 years | 2267 | 185 | 1.18 (0.98-1.43) |
| More than 5 years | 4887 | 330 | 1.23 (1.07-1.42) |

Adjusted for age, sex, country, education, hearing aid use, BMI, tdi, hypertension, stroke, L_den_, PM_2.5_.

Abbreviations: BMI, body mass index; HR, hazard ratio; CI, confidential interval; tdi, townsend deprivation index, L_den_: 24-h traffic noise; PM_2.5_, fine particulate matter with diameter < 2.5 μm.

**Table S11 Characteristics of participants with (N=102,296) and without (N=52,412) lifestyle data**

| **Characteristics, n (%)** | **With lifestyle data (n=102,296)** | **Without lifestyle data (n=52,412)** | ***P* values** |
| --- | --- | --- | --- |
| Age, year | 56.6 ± 8.2 | 56.6 ± 8.2 | 0.279 |
| Male | 47183 (46.1) | 22157 (42.3) | <0.001 |
| Country |  |  | 0.878 |
| England | 100669 (98.4) | 51573 (98.4) |  |
| Wales | 1627 (1.6) | 839 (1.6) |  |
| Education |  |  | <0.001 |
| Higher | 35544 (34.7) | 17226 (32.9) |  |
| Upper secondary | 34370 (33.6) | 16431 (31.3) |  |
| Lower secondary | 5765 (5.6) | 3058 (5.8) |  |
| Vocational | 12237 (11.9) | 5838 (11.1) |  |
| No secondary education | 13752 (13.4) | 8946 (17.1) |  |
| Prefer not to answer | 628 (0.6) | 913 (1.7) |  |
| Hearing aid use | 2860 (2.8) | 1558 (3.0) | <0.001 |
| BMI | 27.2 ± 4.6 | 27.2 ± 4.8 | 0.003 |
| Townsend deprivation index | - 1.3 ± 2.9 | -1.0 ± 3.0 | <0.001 |
| PM_2.5_ | 9.9 ± 0.9 | 9.9 ± 0.9 | <0.001 |
| L_den_ | 55.9 ± 4.2 | 55.9 ± 4.3 | 0.056 |
| Hypertension | 26018 (25.4) | 13483 (25.7) | 0.214 |
| Stroke | 1278 (1.2) | 751 (1.4) | 0.003 |

Data are mean ± SD or frequencies (percentages).

Abbreviations: BMI, body mass index; SD: standard deviation; tdi, townsend deprivation index, L_den_: 24-h traffic noise; PM_2.5_, fine particulate matter with diameter < 2.5 μm.

**Figure S1** **Flowchart of the study**

170,292 participants with accurate information of noise workplace

Excluded: Participants with no accurate information of noisy workplace (N=332,109)

UK Biobank (N=502,401)

154,708 participants for the association of noise in the workplace, PRS with type 2 diabetes

160,257 participants for the association between noise in the workplace and type 2 diabetes

Excluded: Participants with diabetes at baseline (N=10,035)

Excluded: Participants with missing information on genetic data (N=5,549)

102,296 participants for the association of noise in the workplace, lifestyle with type 2 diabetes

Excluded: Participants with missing information on lifestyle data (N=52,412)
